# Flax Fiber Hydrophobic Extract Inhibits Human Skin Cells Inflammation and Causes Remodeling of Extracellular Matrix and Wound Closure Activation

**DOI:** 10.1155/2015/862391

**Published:** 2015-08-04

**Authors:** Monika Styrczewska, Anna Kostyn, Anna Kulma, Grazyna Majkowska-Skrobek, Daria Augustyniak, Anna Prescha, Tadeusz Czuj, Jan Szopa

**Affiliations:** ^1^Faculty of Biotechnology, University of Wroclaw, Przybyszewskiego 63/77, 51-148 Wroclaw, Poland; ^2^Institute of Genetics and Microbiology, Faculty of Biological Sciences, University of Wroclaw, Przybyszewskiego 63/77, 51-148 Wroclaw, Poland; ^3^Linum Foundation, Stablowicka 147/149, 54-066 Wroclaw, Poland; ^4^Institute of Genetics and Microbiology, Department of Pathogen Biology and Immunology, University of Wroclaw, Przybyszewskiego 63/77, 51-148 Wroclaw, Poland; ^5^Department of Food Science and Nutrition, Wroclaw Medical University, Borowska 211, 50-556 Wroclaw, Poland; ^6^Department of Genetics, Plant Breeding and Seed Production, Faculty of Life Sciences and Technology, Wroclaw University of Environmental and Plant Sciences, Plac Grunwaldzki 24A, 53-363 Wroclaw, Poland

## Abstract

Inflammation is the basis of many diseases, with chronic wounds amongst them, limiting cell proliferation and tissue regeneration. Our previous preclinical study of flax fiber applied as a wound dressing and analysis of its components impact on the fibroblast transcriptome suggested flax fiber hydrophobic extract use as an anti-inflammatory and wound healing preparation. The extract contains cannabidiol (CBD), phytosterols, and unsaturated fatty acids, showing great promise in wound healing. In *in vitro* proliferation and wound closure tests the extract activated cell migration and proliferation. The activity of matrix metalloproteinases in skin cells was increased, suggesting activation of extracellular components remodeling. The expression of cytokines was diminished by the extract in a cannabidiol-dependent manner, but *β*-sitosterol can act synergistically with CBD in inflammation inhibition. Extracellular matrix related genes were also analyzed, considering their importance in further stages of wound healing. The extract activated skin cell matrix remodeling, but the changes were only partially cannabidiol- and *β*-sitosterol-dependent. The possible role of fatty acids also present in the extract is suggested. The study shows the hydrophobic flax fiber components as wound healing activators, with anti-inflammatory cannabidiol acting in synergy with sterols, and migration and proliferation promoting agents, some of which still require experimental identification.

## 1. Introduction

Wound healing is a very complicated process consisting of three main stages: local inflammation inhibition, cell proliferation, and tissue remodeling and organization. The first stage limits the whole process, as the inflammatory response inhibits skin cell proliferation and differentiation, mainly due to reactive oxygen species production. Chronic inflammation makes it impossible for the wound to heal, causing permanent pain and the possibility of infection [1]. The key factors of chronic inflammation are cytokines and chemokines produced by skin and activated immune system cells, such as IL1, IL6, or TNF-*α*. Such molecules cause reactive oxygen species production, proliferation and recruitment of lymphocytes and macrophages, and degranulation [2]. Another type of very important signaling molecules of wound healing is growth factors, which activate skin cell proliferation and extracellular matrix components reorganization, by promoting extracellular metalloproteinase production. Fibroblast-keratinocyte cross talk is crucial for these well-balanced wound closure and tissue regeneration elements [3].

Problems with wound healing are associated with several medical disorders, such as diabetes, atherosclerosis, and immunological disorders. As these diseases have become more widespread, chronic wounds have become a serious medical problem as we still lack successful therapy for the treatment of chronic wounds of different origins but of the same local background of constant inflammation.

Trying to fulfill the need for such therapy we previously conducted some preclinical tests of wound dressing made of flax fiber [4]. We chose this material partly due to its high hygroscopic properties, but mainly for the elevated levels of bioactive reactive oxygen species scavenging compounds. Flax fiber is very different from pure cellulosic cotton fiber, as it also contains lignin, pectin, and many smaller molecules attached to these polymers [5, 6]. The results of the preclinical test were very promising, with enhancement of wound closure in almost 75% of cases and over 90% of patients reporting pain relief during the therapy.

One of the scopes of research in our group is the analysis of several fractions of all flax products and waste products also, to identify potentially bioactive compounds. Flax (*Linum usitatissimum* L.) is an annual plant of the temperate climate zone, grown for its fiber and seeds. The products obtained from flax, especially seeds and oil produced from them, have been traditionally considered to be health-promoting food and used, for example, in gastrointestinal and heart diseases. The development of modern molecular biology technologies allowed identification and modification of the production level of some of the flax compounds responsible for health-beneficial properties. These achievements concern polyunsaturated fatty acids [7], lignan complexes [8], carotenoids [9], flavonoids, and phenylpropanoids [10]. All these molecules consist of either oil or the seed itself, while there is still a lack of knowledge about the fiber bioactive components. The preclinical trials of flax fiber application as a wound dressing were therefore accompanied by measurements of known bioactive components of the fiber and identification of some new ones. One of the newly identified compounds, cannabidiol, was very interesting, as it had been found previously only in* Cannabis* plants. Identification was based on the UV absorption spectrum, retention time in ultra performance liquid chromatography (UPLC), and mass spectrometry [11].

Cannabinoids owe their name to the* Cannabis* plant, where they were originally identified [12], but, due to further discoveries of the human endocannabinoid system, nowadays they are defined as molecules that can bind to the cannabinoid receptors. Thus the group can be divided into three types of cannabinoids considering their origin: endocannabinoids (produced by the human body itself, e.g., anandamide, arachidonoyl glycerol, and arachidonoyl dopamine), synthetic cannabinoids (e.g., HU-243, WIN-55,212-2, and CP-55,940), and phytocannabinoids (terpenophenolics of plant origin, e.g., tetrahydrocannabinol, cannabidiol, and cannabinol) [13]. The special properties of plant cannabinoids have been known and used for ages by almost all human cultures. In the* Cannabis* plant these anti-inflammatory and pain-killing molecules are synthesized from the terpene part, geranyl pyrophosphate from the plastidic isoprene pathway, and alkylresorcinol—olivetolic acid from the polyketide pathway [14, 15]. Both pathways and molecules as well as their many analogues are present also in flax metabolism. Furthermore, our very last preliminary analysis also suggested existence of genes in the flax plant that may be responsible for the synthesis of terpenophenolics.

Cannabidiol (CBD), a compound that was identified in flax fiber, is the second, after Δ^9^-tetrahydrocannabinol (THC), most analyzed phytocannabinoid. CBD is a nonpsychoactive cannabinoid, still showing valuable anti-inflammatory and antinociceptive properties. It has been shown to attenuate many inflammation and neurodegeneration related changes in gene expression, such as in Alzheimer disease, cerebral ischemia, or multiple sclerosis, but also connected to rheumatoid arthritis and diabetes [16]. CBD activity, mainly connected to specific receptor activation and influencing CREB and NF*κ*B transcription factors [17], results in inhibition of expression of many cytokines and causes lowering of reactive oxygen species production [18, 19].

Considering further application of the fiber as a wound dressing, but also of the flax-derived CBD itself, as well as other extract components, wider bioactivity studies were necessary. We focused on skin cell inflammation and regeneration, as these are the most important stages in the process of chronic wound healing, playing a crucial role in the local tissue condition. In this study we evaluated the flax fiber extract as a possible wound healing preparation by measuring cell proliferation, migration, inflammatory gene expression, and extracellular metalloproteinase activity. At the same time we tried to determine how many flax fiber extract properties can be attributed to the cannabidiol molecule.

## 2. Materials and Methods

### 2.1. Plant Material

Flax (*Linum usitatissimum cv.* Linola, Variety Registration Office, Plant Health and Production Division, Canadian Food Inspection Agency, registration number 5426) plants grown in the field, harvested 90 days after seeding, were used. Flax fiber was prepared via the standard dew retting process described previously [20]. The fabric was prepared from raw yarn using a standard weaving method. The linear mass of the warp and weft was 140 tex. The warp density was 65/dm^3^ and the linear density of the weft was 85/dm^3^. The density of the final flax fabric was 220 g/m^3^. This fabric made from only mechanically affected fiber was used for extraction of the biologically active compounds.

### 2.2. Preparation of Flax Fiber Hydrophobic Extract

Unbleached linen fabric (15 g) was extracted three times with 50 mL of chloroform, the solutions were pooled, and after drying in a nitrogen atmosphere, the matter was redissolved in 500 *μ*L of ethanol. The extract was filtered through 0.25 *μ*m Acrodisc and the concentration of cannabidiol in the extracts was measured by UPLC analysis in comparison to a commercially available CBD standard (Sigma-Aldrich).

### 2.3. Terpenophenolics UPLC Analysis

The analysis was performed with a Waters Acquity ultra performance liquid chromatograph with a PDA and additional mass detector. The mobile phase was an acetonitrile-water gradient of 70 : 30 for 1 min followed by an acetonitrile gradient from 70% to 100% for 5 min, 100 : 0 until 10 min, and then a 1-min return to 30 : 70 for 1 min (flow 0.4 mL/min^−1^). 0.05% trifluoroacetic acid (TFA) was added to both solvents for elimination of the phenolic compounds tailing. The detection and integration of the cannabidiol peak was performed at 230 nm in comparison to the compound standard (Sigma-Aldrich, 98.5% purity).

### 2.4. GC-FID Analysis of Squalene and Sterols

1 mL of extract was reextracted twice with 10 mL of hexane by vortexing vigorously for 15 min. The extracts were pooled and evaporated under nitrogen (25°C) to dryness. Trimethylsilyl derivatives of sterols were prepared according to the method described by Shukla et al. [21]. The separation of silyl compounds and the quantification were performed on a gas chromatograph 6890 N (Agilent Technologies, USA) equipped with an FID detector and the capillary column HP-5 30 m × 0.32 mm × 0.25 *μ*m (Agilent Technologies, USA). Helium was used as a carrier gas at a flow rate of 1.0 mL/min and the separation was carried out at a temperature set from 250°C (for 5 min) to 290°C (for 14 min); the temperature increased at a rate of 5°C/min. 5*α*-Cholestane was used as an internal standard for quantitative analysis and Chemstation v. B.04.02 was used to calculate the results.

### 2.5. GC-FID Analysis of Fatty Acid Content

#### 2.5.1. Conversion of Fatty Acids into Fatty Acid Methyl Esters

Prior to GC analysis, the fatty acids were converted into their methyl esters (FAME). 5 *μ*L of extract was placed in a glass tube with a Teflon-sealed screw cap and supplemented with 500 g of pentadecanoic acid as an internal standard. Each sample was made in triplicate. 1 mL of 0.5 M KOH in anhydrous methanol was added to each sample, which was shaken well and incubated for 30 min at 70°C. After cooling, 1 mL of 1.25 M HCl in anhydrous methanol was added and the sample was again mixed and incubated at 70°C for 30 min. Samples were cooled, and 1 mL of hexane and 3 mL of saturated NaCl were added to each. Samples were then mixed well for 5 min and the hexane fraction containing FAME was collected in new Eppendorf tubes. Extraction was repeated by adding an additional 1 mL of hexane, and the collected hexane fractions were stored at 4°C until measurement (at the latest on the next day).

#### 2.5.2. FAME Analysis

FAME analysis was carried out using an Agilent 7890A gas chromatograph with an FID detector on a DB-23 column (60 m, 0.25 mm, and 0.25 *μ*m) suitable for the determination of fatty acid content and composition. Each FAME was identified by its retention time, and its quantity was calculated according to an internal standard. Each sample was esterified and measured in 3 replicates. The fatty acid content was measured using the following program: injector temperature: 240°C; split 100 : 1; gas pressure: 17.81 psi; carrier gas: nitrogen; Owen program: 140°C (hold 2 min) to 240°C (4°C/min; hold 10 min); and FID detector temperature: 260°C. Series of fatty acid standards (Sigma-Aldrich) were analyzed to determine the retention times for each compound.

### 2.6. Human Skin Fibroblast and Keratinocyte Culture Conditions

Normal human dermal fibroblasts (NHDF) and normal human epidermal keratinocytes (NHEK) (PromoCell, Germany) were cultivated in sterile conditions in 5% CO_2_ atmosphere and at 37°C temperature. NHDF were grown in DMEM medium containing 1.5 g/L glucose, 10% fetal calf serum, 100 U/mL penicillin, and 100 *μ*g/mL streptomycin until 90% confluence and then trypsinized (0.05% trypsin/1 mM EDTA in calcium- and magnesium-free PBS). Trypsin was inhibited by addition of fresh medium in a 5 : 1 volume ratio; cells were centrifuged at 800 ×g for 5 minutes and further plated to obtain 7 to 10 times area dilution. NHEK cells were grown in KGM 2 medium (Lonza, Germany). For trypsinization Detach Kit (PromoCell, Germany) was used according to the manufacturer's instructions. Cells were centrifuged 220 ×g for 3 minutes and plated to obtain a maximum of 7 times area dilution.

### 2.7. Skin Cell Morphology Observation and Basic Proliferation Tests

For the basic proliferation test NHDF and NHEK cells were cultivated in 24-well plates for 24 hours and after the fresh medium was added, incubated with increasing concentrations of hydrophobic flax fiber fraction. The morphology of the cells was visualized after 24 hours of incubation, using a transmitted light phase contrast microscope equipped with ×10 and ×100 objectives (Axiovert 40 CFL, ZEISS). After that the MTT reagent (Sigma, USA) was added to make a final concentration of 0.4 mg/mL and incubated for 4 hours. Produced formazan crystals were dissolved in 0.5 mL of DMSO for 30 minutes and measured at 540 nm in an Asys UVM340 Microplate Reader (Biochrom, UK). The experiment was performed in four biological replicates.

### 2.8. *In Vitro* Wound Scratch Assay

Wound healing properties were evaluated using the* in vitro* scratch assay [22], which measures the expansion of a cell population on surfaces. Skin cells were cultivated in a 24-well plate for about 48 hours, until nearly confluent monolayers were obtained. Next, the monolayer was linearly disrupted with a sterile 10 *μ*L plastic pipette tip. The cellular debris was removed by washing the culture with PBS, and then fresh medium was added, containing 0.5% fetal calf serum, 100 U/mL penicillin and 100 *μ*g/mL streptomycin in the case of NHDF cells, and KGM 2 medium without pituitary extract for NHEK cells. Then, the flax fiber hydrophobic extracts were added. Nontreated cells served as a control, but also ethanol samples were analyzed. The experiment was performed in four biological replicates. To estimate the relative migration of skin cells, representative images of the scratched areas from each well were photographed using a transmitted light phase contrast microscope equipped with ×10 and ×100 objectives (Axiovert 40 CFL, ZEISS) at zero time and after 6, 12, and 24 hours. The data were analyzed using TScratch software (Computational Science and Engineering Laboratory, Zurich, Switzerland).

### 2.9. Matrix Metalloproteinase Activity Assay

For the treatment, the NHDF or NHEK cells were grown in six-well plates for 24 hours. After fresh medium had been added, the inflammatory state was induced with LPS (100 ng/mL final concentration in the culture) in PBS. The incubation lasted 6 hours, before the flax fiber extract (the final concentration of CBD was 0.2 *μ*g/mL for Ex 1, 0.1 *μ*g/mL for Ex 2, and 0.04 *μ*g/mL for Ex 5) and control samples were added and incubated for 24 hours.

The general activity of matrix metalloproteinase (MMP) enzymes was determined using the MMP Activity Assay Kit (ab112147) from Abcam, according to the manufacturer's protocol. The medium after cell treatment was collected and used for the test. The medium without cell incubation was used as a negative control. The amount of 25 *μ*L of medium was added to 25 *μ*L of 2 mM APMA and incubated for 3 hours at 37°C. After incubation 50 *μ*L of MMP Red Substrate working solution was added to the samples. After one hour of incubation at room temperature in the darkness the fluorescence intensity was measured at Ex/Em = 490/525 nm, using a microplate reader (Varioskan Flash, Thermo Scientific).

### 2.10. Skin Cell Treatment for Gene Expression Analysis

For the treatment, the NHDF or NHEK cells were grown, induced with LPS, and treated analogically to MMP activity evaluation. An ethanol negative control and CBD and *β*-sitosterol standard positive controls were also included (pure CBD in final concentrations corresponding to flax preparations—CBD 1-0.2 *μ*g/mL, CBD 2-0.1 *μ*g/mL, and CBD 5-0.04 *μ*g/mL, and *β*-sitosterol—SIT 1-3.63 *μ*g/mL, SIT2, 1.82 *μ*g/mL, and SIT5-0.73 *μ*g/mL). The ethanol concentration was 0.1%. The cells were harvested after 24 hours of treatment and directly used for RNA isolation.

### 2.11. RNA Isolation, cDNA Synthesis, and Real-Time PCR Reactions

Total RNA was isolated from freshly harvested cells after the treatments using an RNeasy Plus Mini Kit (Qiagen, Germany) according to the manufacturer's instructions. To ensure no genomic DNA contamination, an isolated amount of 2 *μ*g of each RNA sample was treated with DNase I (Fermentas, Lithuania) and directly used as a template for cDNA synthesis. cDNAs were synthesized using a High-Capacity cDNA Reverse Transcription Kit (Applied Biosystems, USA). Real-time PCR reactions were set according to SYBR Green Master Mix (Applied Biosystems, USA) producer's instructions; the primer sequences are presented in Table 1. Obtained data were analyzed using ΔΔCt methodology for calculation of the parameter RQ (relative quantification).

### 2.12. Statistical Analysis

The statistical significance of obtained results was evaluated using Student's* t*-test. All of the calculations were carried out using the STATISTICA 10 software (StatSoft). Values of *P* < 0.05 were considered significant.

## 3. Results and Discussion

### 3.1. Preparation and Characterization of Flax Fiber Cannabinoid Containing Extract

The flax fiber was extracted with chloroform; the solvent was evaporated and the matter was redissolved in ethanol, prior to bioactivity tests. The composition of the extract was determined by HPLC and GC analysis. The main components of the extract are phytosterols, fatty acid, cannabidiol, and the carotenoid lutein. The concentrations of the components are presented in Table 2.

Sterols have previously been identified in flax fiber extracts [23], and their presence was predictable considering their widespread occurrence in plants and the extraction method used. Final concentration of sterols in the preparation was 7.31 mg/mL, and *β*-sitosterol, the most common phytosterol, constituted more than 50% of total sterols.

Phytosterols are well-known biologically active molecules, mainly lowering serum cholesterol and lipid levels. These properties are connected to change in cholesterol incorporation into mixed micelles but also modulation of cholesterol metabolism in the cell through Niemann-Pick C1-like 1 protein and ABC-type transporters, resulting in lower cholesterol intake [24]. They also influence* de novo* cholesterol synthesis by modulating the mevalonic pathway, particularly hydroxymethylglutaryl coenzyme A (HMG CoA) reductase [25]. This reductase and other genes of this pathway were among those most strongly inhibited in the NHDF cells when treated with the flax fiber hydrophobic fraction in whole transcriptome microarray studies.

The extract also contains fatty acids of a final concentration of 3.95 mg/mL, with almost 40% unsaturated fatty acids. The level of the most valuable polyunsaturated fatty acids (PUFA) was 0.524 mg/mL for linoleic and 0.054 mg/mL for linolenic acid (see Suppl. Table  1 in Supplementary Material available online at http://dx.doi.org/10.1155/2015/862391). The content of the extract clearly suggests its positive function in the process of wound healing, with strong antioxidative potential, anti-inflammatory activity of cannabidiol and also sterols [26], and potentially proliferation promoting unsaturated fatty acids [27].

Among fatty acids, especially polyunsaturated ones have already been identified as inducers of wound healing, promoting angiogenesis, antioxidation, and some direct anti-inflammatory actions, mainly inhibiting prostaglandin and leukotriene formation [28, 29]. The presence of PUFA in flax products has been most widely examined, considering bioactive components [30, 31]. Their presence and biological activity were also not the main impact on fibroblast transcriptome identified in previous studies of flax fiber extract, where cannabidiol and possibly sterol characteristic changes were observed.

The CBD content was measured using UPLC analysis in comparison to the pure cannabidiol standard. The concentrated preparation consisted of 0.211 mg/mL CBD. UPLC analysis also revealed the presence of traces of lutein, a carotenoid common in flax fractions.

The presence of sterols and fatty acids in the flax fiber preparation may also be partially responsible for the anti-inflammatory activity, as they are proven inhibitors of cytokine production [26], but the quantitative analysis of microarray data reveals that almost 60% of observed changes caused by the flax fiber hydrophobic extract are related to cannabidiol presence. Still sterols and squalene identified in the extract, as well as fatty acids, can act in synergy, as is the case with many different natural preparations.

### 3.2. Flax Fiber Hydrophobic Extract Influence on Skin Cell Proliferation and Morphology

Fibroblast and keratinocyte cell cross talk plays a crucial role in the wound healing progression. Skin cells are the most important in the main stages of this process: inflammation, producing a plethora of signaling cytokines and chemokines, and tissue regeneration, being responsible for the extracellular matrix production and organization and building the skin layers [2, 3]. For these reasons fibroblasts and keratinocytes have been chosen for flax fiber extract biological activity examinations.

**Figure 1:**
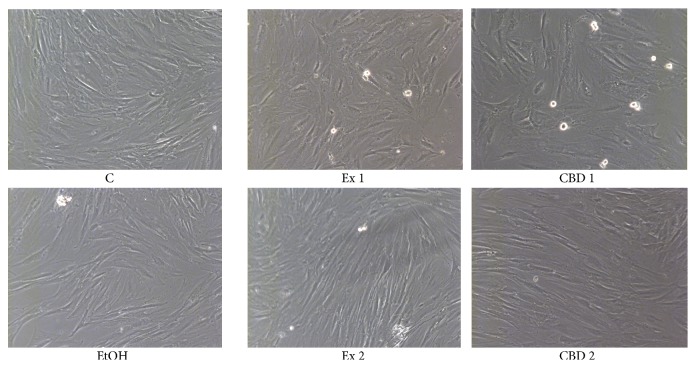
NHDF cell treatments with flax fiber cannabidiol containing extract, morphology observations. C: control untreated cells; EtOH: NHDF cells after 24-hour incubation with ethanol, used as a negative control; Ex 1: cells treated with flax fiber cannabinoid preparation, final CBD concentration 0.211 *μ*g/mL of culture; Ex 2: cells after incubation with preparation diluted twice with ethanol, 0.105 *μ*g CBD/mL of culture; CBD 1 and CBD 2: cells treated with pure CBD standard, final concentrations of 0.211 and 0.105 *μ*g/mL, respectively, used as positive controls.

To examine the basic impact of flax fiber hydrophobic extract on the skin cells, we observed their morphology and ability to metabolize harmful substance (the MTT test) in the presence of the extract. This kind of observation is a widely known measure of cell proliferation potential. These experiments also allowed us to establish the proper range of extract concentration depending on skin cell type. We also included pure CBD samples in the analysis, as there is no information on the influence of this kind of molecule on skin cell cultures, while other possible bioactive components (sterols and fatty acids) do not show any cytotoxicity in the used range of concentrations.  Figure 1 shows representative images of NHDF cells after 24 hours of incubation. We did not observe any changes in fibroblast cell morphology caused by flax fiber extract, even in the highest obtainable concentration of 0.211 *μ*g/mL (Ex 1) nor in the CBD standard used as a positive control.

**Figure 2:**
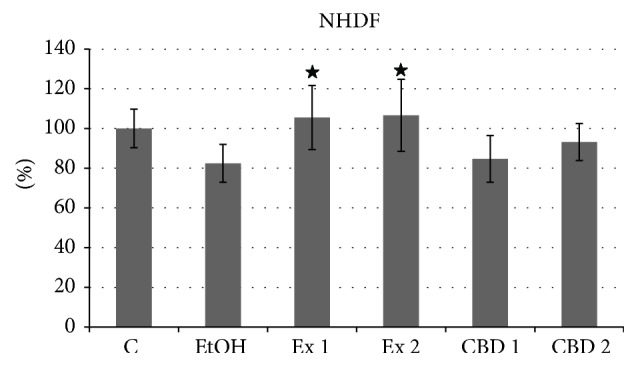
MTT test of NHDF cells treated with flax fiber cannabinoid containing extract. Final CBD concentrations 0.211 (Ex 1) and 0.105 (Ex 2) *μ*g/mL; C: untreated cells; EtOH: ethanol-treated cells, negative control; CBD 1 and 2: positive control samples of pure CBD in concentration corresponding to Ex 1 and 2. Measurements of four biological replicates, ± SD, *P* < 0.05.

Basic proliferation potential tests of human fibroblast cells after incubation with flax cannabinoid preparation revealed a slight positive effect on the cells. The results (presented in Figure 2) suggest that the influence is not connected to CBD presence in the extract, since the positive control sample did not enhance the proliferation potential. The observed influence is statistically significant, yet not related to the extract concentration. A wider range of concentrations will need to be tested in order to establish whether flax fiber extract can influence cell proliferation.

**Figure 3:**
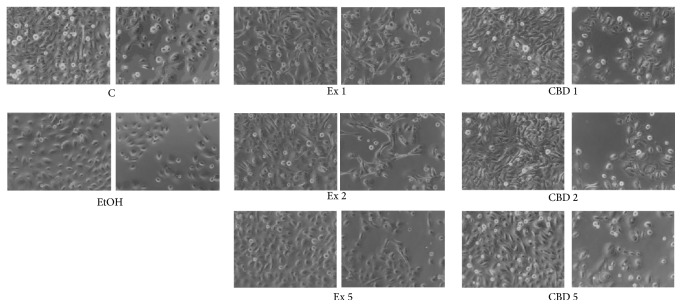
NHEK cells after treatments with flax fiber cannabinoid extract. C: control untreated cells; EtOH: cells after 24-hour incubation with ethanol, the negative control; Ex 1: cells treated with the extract where final CBD concentration was 0.211 *μ*g/mL of culture; Ex 2: cells after incubation with extract diluted twice with ethanol, 0.105 *μ*g CBD/mL of culture; Ex 5: cells incubated with five times diluted Ex 1, CBD concentration 0.042 *μ*g/mL; CBD 1, CBD 2, and CBD 5: cells treated with pure CBD standard, final concentration analogical to Ex 1, 2, and 5, respectively, used as positive controls. Images on the left side show the cells of each sample growing in a large confluence, while the ones on the right represent the same sample but in a region of lower growth density.

Analogical experiments were also conducted for keratinocytes, but due to their known sensitivity a lower extract concentration (Ex 5: final CBD conc. 0.042 *μ*g/mL) was also included. The first treatments of NHEK cells with the flax fiber hydrophobic extract showed a slightly negative effect of the highest extract concentration both in morphology of the culture and in proliferation potential. Observations of NHEK cells treated with Ex 1 demonstrated a greater amount of unattached cells and cell debris than in other samples, but the difference was not very dramatic. What was clearly seen was the influence of the extract on the shape of the keratinocytes (Figure 3). In the presence of fiber extract NHEK cells appeared longer and more spindle-like, with more protrusions. This kind of cell shape change is most often associated with increased motility. The changes were extract concentration-dependent, which was especially apparent in regions of lower confluence (images on the right in Figure 3), but no similar changes were observed in the corresponding positive control pure CBD samples. These observations suggest that flax fiber hydrophobic extract changes the cell shape of keratinocytes and possibly their motility, but it is not the CBD itself that is responsible for that influence.

**Figure 4:**
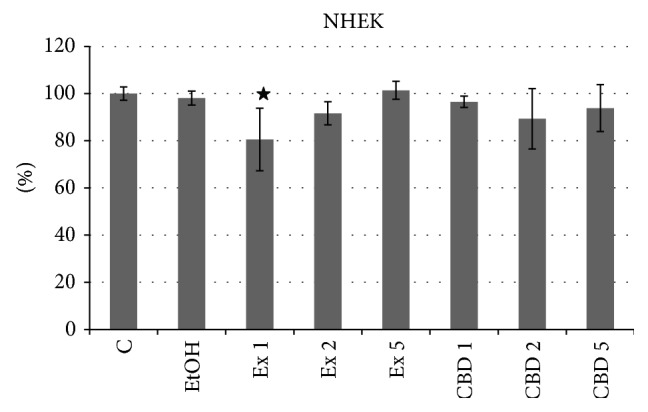
MTT test of NHEK cells treated with flax fiber cannabinoid extract. Final CBD concentrations 0.211 (Ex 1), 0.105 (Ex 2), and 0.042 (Ex 5) *μ*g/mL; C: untreated cells; EtOH: ethanol-treated cells used as negative control; CBD 1, 2, and 5: positive control samples of pure CBD in concentration corresponding to Ex 1, 2, and 5. Measurements of four biological replicates, ± SD, *P* < 0.05.

The observations of morphological changes were complemented by the basic proliferation potential test that confirmed some negative influence of the extract at the highest concentration on the NHEK cells (Figure 4). No such decrease was observed with pure CBD; therefore it can be assumed that the compound that is responsible for that effect is not the flax cannabidiol, but some other component of the extract.

### 3.3. Flax Fiber Hydrophobic Extract Influence on Skin Cell Wound Closure

**Figure 5:**
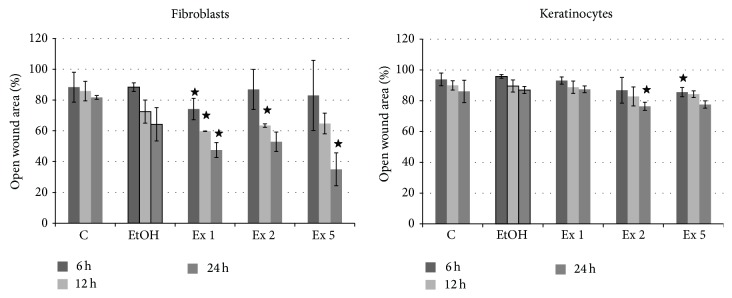
Flax fiber hydrophobic extract influence on wound closure, “wound scratch assay.” Final CBD concentrations 0.211 (Ex 1), 0.105 (Ex 2), and 0.042 (Ex 5) *μ*g/mL; C: untreated cells; EtOH: ethanol-treated cells used as a negative control. Measurements of four biological replicates, ± SD, *P* < 0.05.

The process of skin cell migration is one of the most important phases of wound healing. It is closely connected to proliferation, extracellular matrix remodeling, expression of adhesion molecules and growth factors, and morphological changes of the cells. These types of alterations have been reported above but also in previous microarray analysis, as caused by flax fiber cannabidiol containing extract. The simple migration tests, “wound scratch assays,” performed on skin cells revealed that the extract in fact influences this process. The results (shown in Figure 5) indicate a slight response of the keratinocytes, but stronger migration activation in fibroblasts, noticeable even after 6 hours in the presence of the highest extract concentration. Nevertheless, migration was activated in both types of skin cells.

Considering components of the extract, plant sterols, in particular *β*-sitosterol, were shown to influence skin cell migration and proliferation. The* Aloe vera* derived *β*-sitosterol was shown to have a proangiogenic and migration-promoting impact on human endothelial cells [32], while *β*-sitosterol from* Rhaponticum carthamoides* (Willd.) Iljin showed a positive effect on keratinocyte cell line proliferation [33]. Since in the case of a “wound scratch assay” type of experiment the effect of migration and proliferation cannot be truly distinguished, the wound closure observed may be a sum of these processes, as it is* in vivo*. The relatively high concentration of *β*-sitosterol in the flax fiber hydrophobic extract may be responsible therefore for the observed results of the* in vitro* wound closure test. The actual role of this component in the process was investigated in further analysis.

### 3.4. Changes in MMP Activity Caused by Flax Fiber Hydrophobic Extract Components

**Figure 6:**
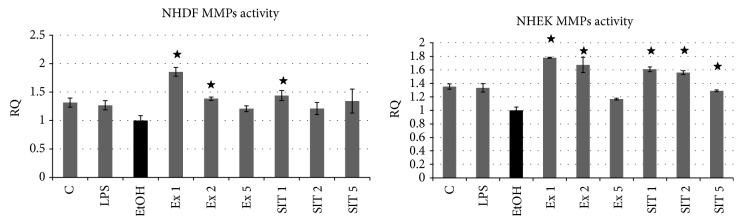
Influence of flax fiber hydrophobic extract on skin cell matrix metalloproteinase activity. Final *β*-sitosterol concentrations in flax extracts: 3.63 (Ex 1), 1.82 (Ex 2), and 0.73 (Ex 5) *μ*g/mL; C: untreated cells; EtOH: ethanol-treated cells used as negative control; SIT1, SIT2, and SIT5: positive control samples of pure *β*-sitosterol in concentrations corresponding to Ex 1, 2, and 5. Measurements of three replicates, ± SD, *P* < 0.05.

Extracellular matrix remodeling is of great importance in the process of wound healing, as it determines the tissue structure and cohesion. This process is closely connected to skin cell migration and proliferation, the two main mechanisms involved in skin regeneration. Matrix metalloproteinases play a crucial role in the remodeling of extracellular components, which is a key factor of skin cell migration and wound closure. Therefore the flax fiber hydrophobic component biological activity analysis was expanded to MMP activity measurements, performed after flax extract treatments of the cells. *β*-Sitosterol control samples were also included in the analysis due to mentioned properties of migration and proliferation activation. The obtained results are shown in Figure 6.

Flax fiber hydrophobic components activated the matrix metalloproteinase activity in both types of skin cells in a concentration-dependant manner. Similar activation was also observed in *β*-sitosterol positive control samples, which suggests that flax fiber phytosterols may be responsible for the MMP activation. The influence of this kind of molecule on the process of extracellular matrix remodeling was previously reported in the aspect of fibrosis [34].

### 3.5. Influence of Flax Fiber Hydrophobic Extract on Expression of Extracellular Matrix Associated Genes

**Figure 7:**
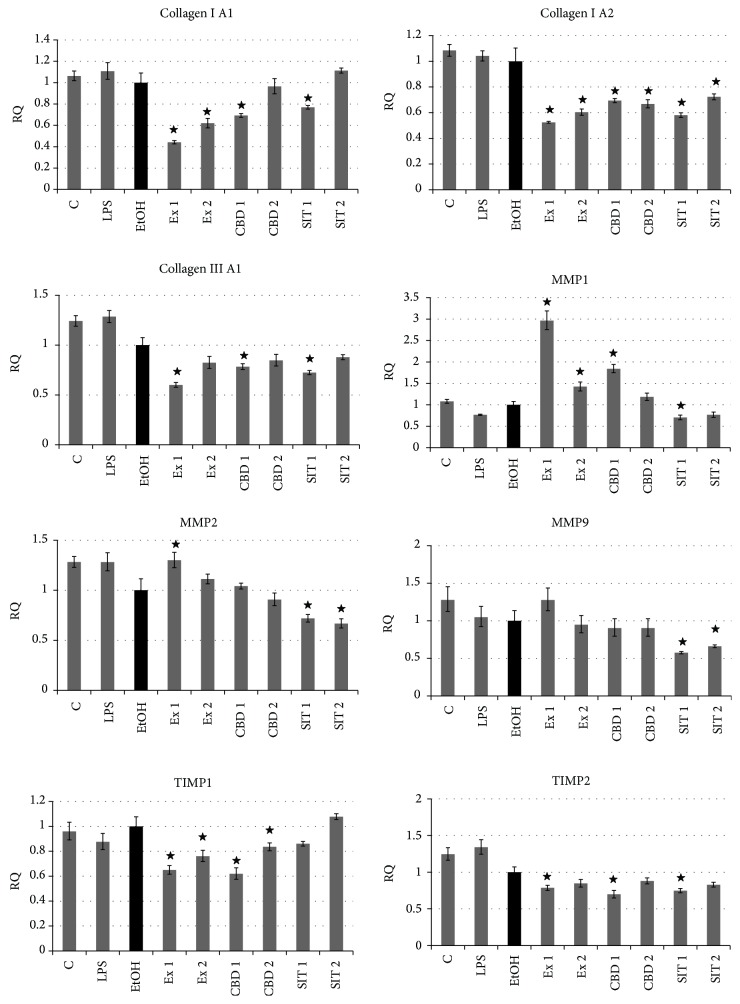
Expression of genes related to extracellular matrix in NHDF cells after treatments with flax fiber hydrophobic extract. Final CBD concentrations 0.211 (Ex 1) and 0.105 (Ex 2) *μ*g/mL; C: untreated cells; EtOH: ethanol-treated cells used as negative control; CBD 1 and 2: positive control samples of pure CBD in concentration corresponding to Ex 1 and 2; SIT1 and SIT2: positive control samples of pure *β*-sitosterol in concentration corresponding to Ex 1 and 2 (3.63 and 1.82 *μ*g/mL). Measurements of five replicates, ± SE, *P* < 0.05.

**Figure 8:**
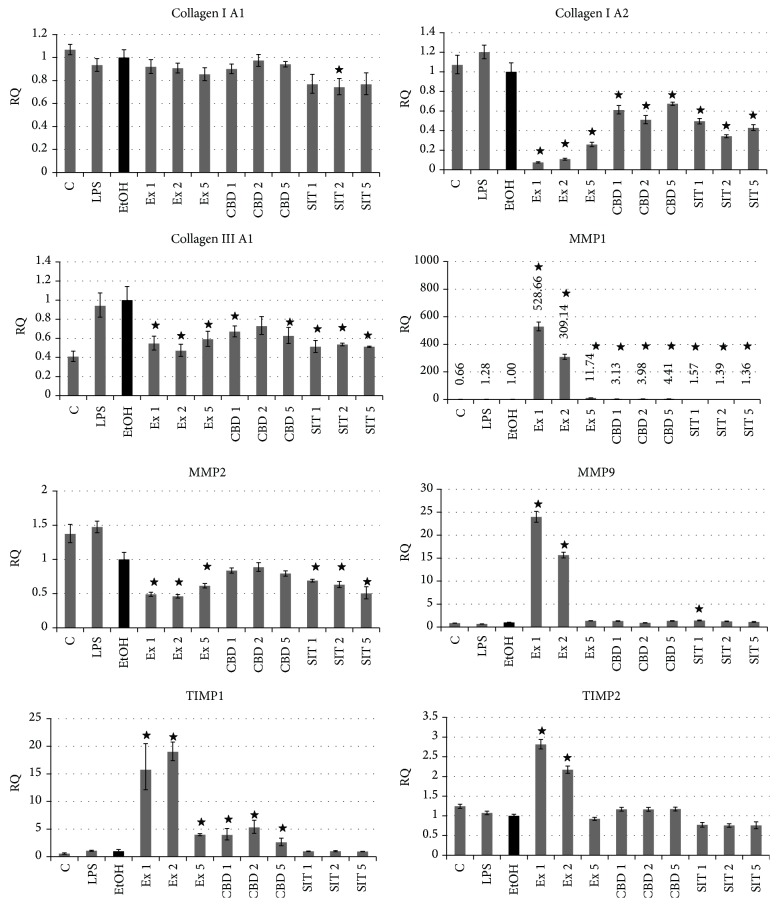
Expression of genes related to extracellular matrix in NHEK cells after treatments with flax fiber cannabinoid containing extract. Final CBD concentrations 0.211 (Ex 1), 0.105 (Ex 2), and 0.042 (Ex 5) *μ*g/mL; C: untreated cells; EtOH: ethanol-treated cells used as negative control; CBD 1, 2, and 5: positive control samples of pure CBD in concentration corresponding to Ex 1, 2, and 5; SIT1, SIT2, and SIT5: positive control samples of pure *β*-sitosterol in concentrations corresponding to Ex 1, 2, and 5 (3.63, 1.82, and 0.73 *μ*g/mL). Measurements of five replicates, ± SE, *P* < 0.05.

The previously reported experiments with microarrays indicated that the flax fiber cannabidiol containing extract may also show an inhibiting influence on the expression of extracellular matrix components, while it activated the production of their regulators—metalloproteinases and their inhibitors [11]. The observed changes in keratinocyte morphology,* in vitro* wound closure tests, and MMP activity induction also indicate extracellular structure remodeling. These preliminary results prompted further expression investigation after induction of the inflammatory state to mimic the chronic wound environment and flax fiber hydrophobic extract treatments, performed with RT real-time PCR. CBD and *β*-sitosterol controls were included, as both substances may be connected to expression modulation, as suggested by microarray and MMP activity studies. The results are presented in Figures 7 and 8 for fibroblasts and keratinocytes, respectively.

Expression analysis revealed inhibition of collagen production, both in fibroblast and in keratinocyte cells, caused by flax fiber hydrophobic extract. Comparable inhibition was also observed for CBD and *β*-sitosterol positive controls in most cases, not always relative to respective extract concentrations (collagen I A2 in keratinocytes). This type of change suggests synergy in extract component activity.

In both types of skin cells the hydrophobic extract caused activation of proteinases regulating the level of extracellular matrix components. In the case of keratinocytes, the activation was extremely large, even over 500 times the MMP1 transcript level in relation to the ethanol control. MMP1 is more specific for fibroblast cells, where its expression was also elevated, more inducible in keratinocytes, and responsible for collagens I, II, and III degradation in the process of cell migration [35]. Its activation has been shown to be connected with IL1*β* signaling [36], which also showed increased expression in this type of cell in further investigation of genes related to inflammation. The changes observed for metalloproteinases are only partially correlated with the cell response to pure CBD, showing the same direction, but not the level of alterations as the ones caused by flax fiber hydrophobic extract. The effect of *β*-sitosterol was of even lower intensity than CBD. This suggests their synergistic action or participation of other components of flax extract in the activation of matrix metalloproteinases.

The expression analysis of tissue inhibitors of metalloproteinases (TIMPs) shows different nature of fibroblast and keratinocyte cells' response. The inhibition observed in fibroblasts (fully correlated with CBD, but only partially with *β*-sitosterol concentration), together with the decrease in collagen production and activation of MMP expression, indicates catabolic changes in extracellular matrix component turnover. Such changes are responsible for looseness of the matrix structure, making migration and tissue remodeling easier. The activation of TIMP production observed in keratinocytes, together with high MMP expression activation and collagen production inhibition, also suggests remodeling of the matrix components, but not of such catabolic orientation as in the case of fibroblasts. Nevertheless, the previous MMP activity studies show an increase of their activity in both types of cells.

The analysis of extracellular matrix related genes also suggests the presence of bioactive components of the extract other than cannabidiol and *β*-sitosterol, which can influence expression of these genes. The response of fibroblasts is more strongly correlated with the CBD concentration than changes observed in keratinocytes. The synergistic action of both components also cannot be excluded. In the case of natural plant extracts, the role of “background components” in biological activity is often observed. The presence of even small amounts of other compounds dramatically influences the activity of the main active components [37, 38]. Such an “entourage effect” cannot be excluded also in the case of used flax extracts.

### 3.6. Flax Fiber Hydrophobic Extract's Influence on Expression of Inflammation-Related Genes in Skin Cells

**Figure 9:**
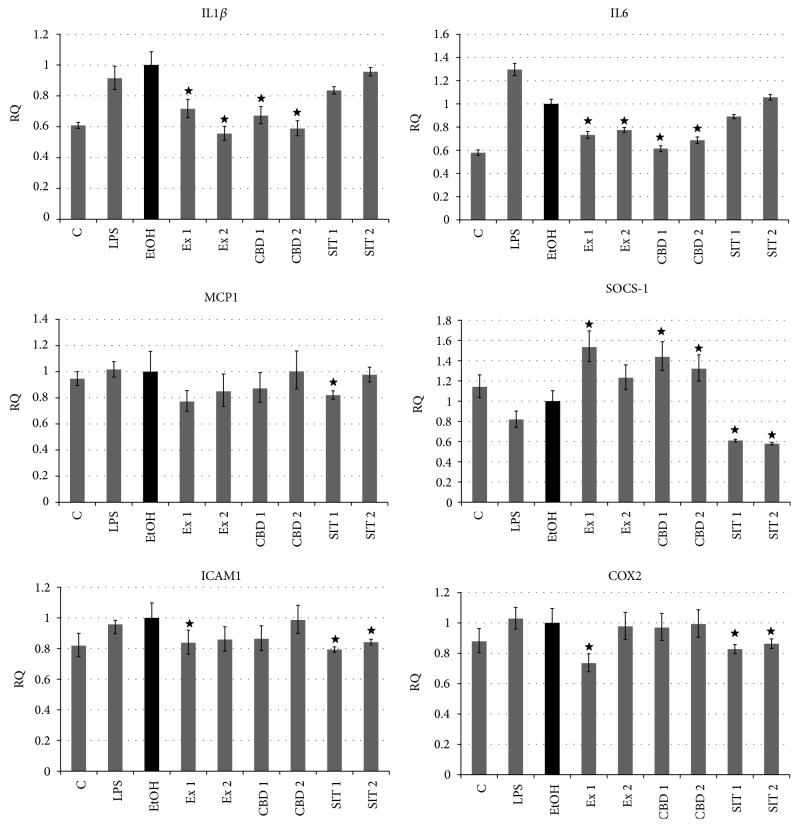
RT real-time PCRs of genes related to inflammatory state expressed by NHDF cells after treatment with flax fiber hydrophobic extract. Final CBD concentrations 0.211 (Ex 1) and 0.105 (Ex 2) *μ*g/mL; C: untreated cells; EtOH: ethanol-treated cells used as negative control; CBD 1 and 2: positive control samples of pure CBD in concentration corresponding to Ex 1 and 2; SIT1 and SIT2: positive control samples of pure *β*-sitosterol in concentration corresponding to Ex 1 and 2 (3.63 and 1.82 *μ*g/mL). Measurements of five replicates, ± SE, *P* < 0.05.

**Figure 10:**
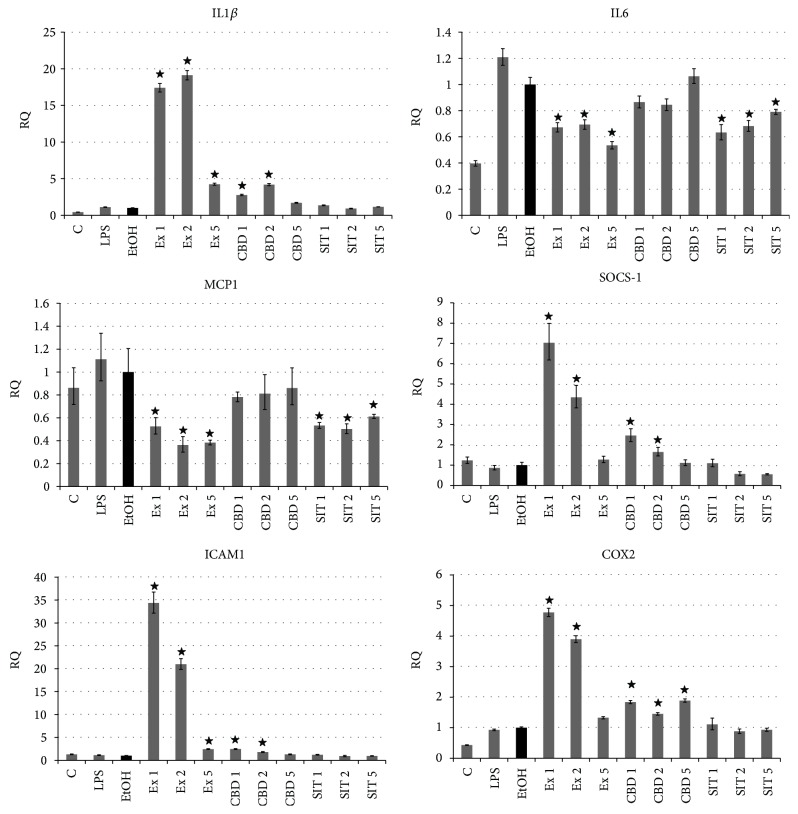
Expression of inflammation-related genes in NHEK cells after treatment with flax fiber cannabinoid extract. Final CBD concentrations 0.211 (Ex 1), 0.105 (Ex 2), and 0.042 (Ex 5) *μ*g/mL; C: untreated cells; EtOH: ethanol-treated cells used as negative control; CBD 1, 2, and 5: positive control samples of pure CBD in concentration corresponding to Ex 1, 2, and 5; SIT1, SIT2, and SIT5: positive control samples of pure *β*-sitosterol in concentrations corresponding to Ex 1, 2, and 5 (3.63, 1.82, and 0.73 *μ*g/mL). Measurements of five replicates, ± SE, *P* < 0.05.

Among flax fiber hydrophobic extract components, in particular cannabidiol is a well-known anti-inflammatory agent proposed to be responsible for most of the extract-induced expression changes. Its properties are mainly lowering the expression of proinflammatory genes and increasing the anti-inflammatory ones, by influencing activation of transcription factors of G-protein related pathways. The expression of genes from these pathways was shown to be lowered by flax cannabinoids in previous microarray studies. The inflammation-related genes were also strongly affected by inhibition of proinflammatory genes and activation of genes with the opposite function. The general data from microarrays require confirmation by more precise investigation to confirm and specify the activity of flax-derived cannabinoid. Therefore the expression of crucial genes of inflammation state propagation was analyzed using the RT real-time PCR method. *β*-Sitosterol control samples were also included in the analysis, as this type of component can also play a role in inflammation propagation [39, 40]. The results of this analysis are shown in Figures 9 and 10 for NHDF and NHEK, respectively.

The results of real-time PCR showed inhibition of proinflammatory gene expression in skin fibroblasts, caused by flax extract that is statistically significant in most cases (interleukins 1*β* and 6, cyclooxygenase 2). The extract also significantly activated the expression of suppressor of cytokine and chemokine signaling 1, which is a known anti-inflammatory gene that influences the cell response to both exogenous (bacterial LPS) and endogenous or secondary (TNF-*α*) stimuli at the signaling level. Changes in expression of other genes, though not always statistically significant, show the same direction of extract activity, lowering migration, and adhesion of leukocytes (MCP1 and ICAM1). What is very important is that observed changes correlate with the cannabidiol concentration in the extract, as similar effects were observed for the pure CBD standard. In the case of ICAM and COX2, the results observed for the extract may be connected to presence of phytosterols, as indicated by the effects observed for pure *β*-sitosterol samples. Flax sterols may also be partially responsible for other observed changes, such as in IL1*β*, IL6, or MCP1, possibly acting in synergy with cannabidiol.

The influence of flax fiber hydrophobic extract on the level of inflammation-related gene expression was also investigated in dermal keratinocytes, as, together with fibroblasts, these cells are responsible for local cytokine production in the skin. The results of RT real-time PCR (shown in Figure 10) showed a slightly different response in comparison to fibroblasts. The main cyto- and chemokine transcript levels of this type of cell were lowered (IL6 and MCP1) and the suppressor of their signaling (SOCS-1) expression was significantly elevated, probably as a result of the synergistic effect of CBD and sterols, where similar changes were noted. Still we have observed induction of transcription of some proinflammatory molecules (IL1*β*, ICAM1, and COX2). This induction is not related to the cannabinoid or sterol content of the extract, as it is present only in CBD positive controls, but at a much lower level. The elevated expression of some proinflammatory agents may be connected to the cytotoxic effect of the extract, reported in the proliferation potential test, and at the transcription level it can be visible even in the presence of diluted extract. Still the main inflammation-related genes of keratinocytes indicate inhibition of the inflammation propagation process. The changes are clear when caused by flax fiber extract, but the influence of CBD and *β*-sitosterol is visible, but not at the same level. Such changes again suggest synergistic anti-inflammatory activity of these components.

Observed expression changes are in a large part characteristic of cannabidiol activity. CBD has been widely proven to lower the activity of CREBP- and NF*κ*B-type transcription factors, therefore inhibiting the transcription of genes responsible for inflammation propagation [17, 41]. This type of activity is very valuable in the chronic inflammation state of the wound, where production of the propagation signal is constant or deregulated. While the changes observed in fibroblast cells are more consistent and correlated with the observed alteration for CBD positive controls, the keratinocytes only partially respond in a similar way. The genes responsible for propagation of the inflammation signal remain lowered, but not fully CBD or *β*-sitosterol concentration-dependent. This suggests synergistic anti-inflammatory activity of extract components. Some observed differences of proinflammatory nature in keratinocytes may be connected to the slight cytotoxic effect of the extract or to its other components, as these changes were not correlated with the CBD or *β*-sitosterol concentration. The role of some analyzed genes in the skin cells may also be different than in other tissues, as in the case of IL1*β*—the migration and extracellular matrix remodeling inducer in keratinocytes [36]. The explanation in both cases is nevertheless not possible without further experiments on this matter.

## 4. Conclusions

Flax fiber hydrophobic extract contains many valuable components that can promote wound healing. Their synergistic activity is able to stimulate the limiting stages of the wound healing process, inhibiting chronic inflammation and promoting extracellular matrix remodeling and skin cell migration and/or proliferation. In this work we have proven that flax fiber extract can be part of a formulation to treat long-standing nonhealing wounds. At the same time we confirmed that the anti-inflammatory and collagen production promotion properties of the extract are mainly due to the CBD content (in most cases acting in synergy with *β*-sitosterol), while the effect on matrix remodeling activity is mostly dependent on the phytosterol content. The role of other extract components (e.g., polyunsaturated fatty acids), indicated by the literature as possibly responsible for some anti-inflammatory but mostly migration and proliferation promoting action, requires further experimental elucidation.

## Supplementary Material

The measurements of fatty acid content were conducted with GC-FID and the obtained results concerning the type and the level of compounds are quite similar to the ones already published for the flax fibers. The presence of polyunsaturated fatty acids in the extract is especially important considering wound healing process. These molecules are proven anti-inflammatory agents, influencing production of lipid mediators of the inflammation, and potential activators of skin cells proliferation.

## Figures and Tables

**Table 1:** Primer sequences used in RT real-time PCR.

Gene	Forward primer	Reverse primer
GAPDH	AGGTCGGAGTCAACGGAT	TCCGGAAGATGGTGATG
SOCS-1	TTTTCGCCCTTAGCGTGAAG	CATCCAGGTGAAAGCGGC
IL6	CCAGGAGCCCAGCTATGAAC	CCCAGGGAGAAGGCAACTG
MCP1	CCCCAGTCACCTGCTGTTAT	AGATCTCCTTGGCCACAATG
IL1*β*	TGAACTGAAAGCTCTCCACC	CTGATGGACCAGTTGGGGAA
ICAM1	GCCGGCCAGCTTATACACAA	CAATCCCTCTCGTCCAGTCG
COX2	TTACAATGCTGACTATGGCTACAA	CTTTGACACCCAAGGGAG
COLIA1	GGGCAAGACAGTGATTGAATA	ACGTCGAAGCCGAATTCCT
COLIA2	TCTCTACTGGCGAAACCTGTA	TCCTAGCCAGACGTGTTTCTT
COLIIIA1	CGCTCTGCTTCATCCCACTAT	CGGATCCTGAGTCACAGACAC
MMP1	GTTCCAAAATCCTGTCC	CGTGTAGCGCATTCTGTCC
MMP2	AGATCTTCTTCTTCAAGGACCGGTT	GGCTGGTCAGTGGCTTGGGGTA
MMP9	GGCCACTACTGTGCCTTTGAG	GATGGCGTCGAAGATGTTCAC
TIMP1	CACCCACAGACGGCCTTATGCAAT	AGTGTAGGTCTTGGTGAAGCC
TIMP2	CTCGCTGGACGTTGGAGGAAAGAA	AGCCCATCTGGTACCTGTGGTTCA

**Table 2:** Concentration of identified components of flax fiber hydrophobic extract.

Compound	mg/mL
*β*-Sitosterol	3,627
Saturated fatty acids	2,430
Unsaturated fatty acids	1,520
Campesterol	1,245
4,4-Dimetylsterol	0,985
Awenasterol	0,581
4-Monomethylsterol	0,268
Cycloartenol	0,263
**Cannabidiol**	**0,211**
Squalene	0,176
Campestanol	0,141
Stigmasterol	0,103
Ergosterol	0,100
Lutein	0,013

## References

[1] Kondo T., Ishida Y. (2010). Molecular pathology of wound healing. *Forensic Science International*.

[2] Albanesi C., Pastore S. (2010). Pathobiology of chronic inflammatory skin diseases: interplay between keratinocytes and immune cells as a target for anti-inflammatory drugs. *Current Drug Metabolism*.

[3] Werner S., Krieg T., Smola H. (2007). Keratinocyte-fibroblast interactions in wound healing. *Journal of Investigative Dermatology*.

[4] Skórkowska-Telichowska K., Zuk M., Kulma A. (2010). New dressing materials derived from transgenic flax products to treat long-standing venous ulcers—a pilot study. *Wound Repair and Regeneration*.

[5] Morvan C., Andème-Onzighi C., Girault R., Himmelsbach D. S., Driouich A., Akin D. E. (2003). Building flax fibres: more than one brick in the walls. *Plant Physiology and Biochemistry*.

[6] Preisner M., Wojtasik W., Kulma A., Żuk M., Szopa J. (2014). Flax fiber. *Kirk-Othmer Encyclopedia of Chemical Technology*.

[7] Zuk M., Prescha A., Stryczewska M., Szopa J. (2012). Engineering flax plants to increase their antioxidant capacity and improve oil composition and stability. *Journal of Agricultural and Food Chemistry*.

[8] Prasad K. (2005). Hypocholesterolemic and antiatherosclerotic effect of flax lignan complex isolated from flaxseed. *Atherosclerosis*.

[9] Fujisawa M., Misawa N. (2010). Enrichment of carotenoids in flaxseed by introducing a bacterial phytoene synthase gene. *Methods in Molecular Biology*.

[10] Zuk M., Kulma A., Dymińska L. (2011). Flavonoid engineering of flax potentiate its biotechnological application. *BMC Biotechnology*.

[11] Styrczewska M., Kulma A., Ratajczak K., Amarowicz R., Szopa J. (2012). Cannabinoid-like anti-inflammatory compounds from flax fiber. *Cellular and Molecular Biology Letters*.

[12] Gaoni Y., Mechoulam R. (1964). Isolation, structure, and partial synthesis of an active constituent of hashish. *Journal of the American Chemical Society*.

[13] Hazekamp A. (2008-2009). *Cannabis Review*.

[14] Fellermeier M., Eisenreich W., Bacher A., Zenk M. H. (2001). Biosynthesis of cannabinoids: incorporation experiments with ^13^C-labeled glucoses. *European Journal of Biochemistry*.

[15] Fellermeier M., Zenk M. H. (1998). Prenylation of olivetolate by a hemp transferase yields cannabigerolic acid, the precursor of tetrahydrocannabinol. *FEBS Letters*.

[16] Mechoulam R., Peters M., Murillo-Rodriguez E., Hanuš L. O. (2007). Cannabidiol—recent advances. *Chemistry & Biodiversity*.

[17] Kozela E., Pietr M., Juknat A., Rimmerman N., Levy R., Vogel Z. (2010). Cannabinoids delta(9)-tetrahydrocannabinol and cannabidiol differentially inhibit the lipopolysaccharide-activated NF-kappaB and interferon-beta/STAT proinflammatory pathways in BV-2 microglial cells. *The Journal of Biological Chemistry*.

[18] Esposito G., de Filippis D., Maiuri M. C., de Stefano D., Carnuccio R., Iuvone T. (2006). Cannabidiol inhibits inducible nitric oxide synthase protein expression and nitric oxide production in *β*-amyloid stimulated PC12 neurons through p38 MAP kinase and NF-*κ*B involvement. *Neuroscience Letters*.

[19] Fouad A. A., Al-Mulhim A. S., Jresat I. (2012). Cannabidiol treatment ameliorates ischemia/reperfusion renal injury in rats. *Life Sciences*.

[20] Wróbel-Kwiatkowska M., Zebrowski J., Starzycki M., Oszmiański J., Szopa J. (2007). Engineering of PHB synthesis causes improved elastic properties of flax fibers. *Biotechnology Progress*.

[21] Shukla V. K. S., Dutta P. C., Artz W. E. (2002). Camelina oil and its unusual cholesterol content. *Journal of the American Oil Chemists' Society*.

[22] Gebäck T., Schulz M. M. P., Koumoutsakos P., Detmar M. (2009). TScratch: a novel and simple software tool for automated analysis of monolayer wound healing assays. *BioTechniques*.

[23] Marques G., Rencoret J., Gutiérrez A., del Río J. C. (2010). Evaluation of the chemical composition of different non-woody plant fibers used for pulp and paper manufacturing. *The Open Agriculture Journal*.

[24] Wang D. Q.-H. (2007). Regulation of intestinal cholesterol absorption. *Annual Review of Physiology*.

[25] Mo H., Elson C. E. (2004). Studies of the isoprenoid-mediated inhibition of mevalonate synthesis applied to cancer chemotherapy and chemoprevention. *Experimental Biology and Medicine*.

[26] Othman R. A., Moghadasian M. H. (2011). Beyond cholesterol-lowering effects of plant sterols: clinical and experimental evidence of anti-inflammatory properties. *Nutrition Reviews*.

[27] Elahi N., Mojdeh S., Poordad A. (2012). The effect of fish oil on improvement of first stage bed sore. *Iranian Journal of Nursing and Midwifery Research*.

[28] Bilal S., Haworth O., Wu L., Weylandt K. H., Levy B. D., Kang J. X. (2011). Fat-1 transgenic mice with elevated omega-3 fatty acids are protected from allergic airway responses. *Biochimica et Biophysica Acta*.

[29] Weylandt K. H., Chiu C.-Y., Gomolka B., Waechter S. F., Wiedenmann B. (2012). Omega-3 fatty acids and their lipid mediators: towards an understanding of resolvin and protectin formation. *Prostaglandins and Other Lipid Mediators*.

[30] Ogborn M. R., Nitschmann E., Bankovic-Calic N., Weiler H. A., Aukema H. (2002). Dietary flax oil reduces renal injury, oxidized LDL content, and tissue n-6/n-3 FA ratio in experimental polycystic kidney disease. *Lipids*.

[31] Truan J. S., Chen J.-M., Thompson L. U. (2010). Flaxseed oil reduces the growth of human breast tumors (MCF-7) at high levels of circulating estrogen.. *Molecular Nutrition & Food Research*.

[32] Moon E.-J., Lee Y. M., Lee O.-H. (1999). A novel angiogenic factor derived from Aloe vera gel: *β*-sitosterol, a plant sterol. *Angiogenesis*.

[33] Biskup E., Golebiowski M., Borsuk K., Stepnowski P., Lojkowska E. (2009). Analysis of Rhaponticum carthamoides (Willd.) Iljin crude extracts composition and ability to simulate cell proliferation. *Planta Medica*.

[34] Kim K.-S., Yang H., Lee J. (2014). Effects of *β*-sitosterol derived from *Artemisia capillaris* on the activated human hepatic stellate cells and dimethylnitrosamine-induced mouse liver fibrosis. *BMC Complementary and Alternative Medicine*.

[35] Pilcher B. K., Dumin J. A., Sudbeck B. D., Krane S. M., Welgus H. G., Parks W. C. (1997). The activity of collagenase-1 is required for keratinocyte migration on a type I collagen matrix. *Journal of Cell Biology*.

[36] Wan Y., Belt A., Wang Z., Voorhees J., Fisher G. (2001). Transmodulation of epidermal growth factor receptor mediates IL-1 beta-induced MMP-1 expression in cultured human keratinocytes. *International Journal of Molecular Medicine*.

[37] Ben-Shabat S., Fride E., Sheskin T. (1998). An entourage effect: inactive endogenous fatty acid glycerol esters enhance 2-arachidonoyl-glycerol cannabinoid activity. *European Journal of Pharmacology*.

[38] Wong A. M., Zhang Y., Kesler K. (2010). Genomic and in vivo evidence of synergy of a herbal extract compared to its most active ingredient: rabdosia rubescens vs. oridonin. *Experimental and Therapeutic Medicine*.

[39] Loizou S., Lekakis I., Chrousos G. P., Moutsatsou P. (2010). *β*-Sitosterol exhibits anti-inflammatory activity in human aortic endothelial cells. *Molecular Nutrition and Food Research*.

[40] Mahajan S. G., Mehta A. A. (2011). Suppression of ovalbumin-induced Th2-driven airway inflammation by *β*-sitosterol in a guinea pig model of asthma. *European Journal of Pharmacology*.

[41] Li K., Feng J.-Y., Li Y.-Y. (2013). Anti-inflammatory role of cannabidiol and O-1602 in cerulein-induced acute pancreatitis in mice. *Pancreas*.

