# Supplementary material for: Flax Fiber Hydrophobic Extract Inhibits Human Skin Cells Inflammation and Causes Remodeling of Extracellular Matrix and Wound Closure Activation
Source: Biomed Res Int. 2015 Aug 4;2015:862391. doi: 10.1155/2015/862391 (PMC4539444; doi:10.1155/2015/862391)
Supplement: Supplementary file 1 — The measurements of fatty acid content were conducted with GC-FID and the obtained results concerning the type and the level of compounds are quite similar to the ones already published for the flax fibers. The presence of polyunsaturated fatty acids in the extract is especially important considering wound healing process. These molecules are proven anti-inflammatory agents, influencing production of lipid mediators of the inflammation, and potential activators of skin cells proliferation. [file 862391.f1.pdf]

Supl. Tab. 1. Fatty acid content of the flax fiber hydrophobic extract.

Fatty acid	mg/ml	SD
Palmitic acid (C16:0)	1,256137	0,081443
Stearic acid (C18:0)	0,567735	0,023902
Oleic acid (C18:1)	0,937371	0,071147
Linoleic acid (C18:2)	0,524228	0,050684
Linolenic acid (C18:3)	0,053752	0,032993
Arachidic actid (C20:0)	0,150098	0,007229
Behenic acid (C22:0)	0,149498	0,013821
Tricosanoic acid (C23:0)	0,112541	0,017819
Lignoceric acid (C24:0)	0,200285	0,029965
